# Design and Focus Test of a Preconsultation Decision Aid for Breast Cancer Reconstruction Patients: A Quality Improvement Initiative

**Published:** 2015-06-22

**Authors:** Kenneth J. Hui, Xiang X. Liu, Anna Luan, Gordon K. Lee

**Affiliations:** ^a^Division of Plastic and Reconstructive Surgery, Stanford University Medical Center, Stanford, Calif; ^b^Division of Plastic and Reconstructive Surgery, First Affiliated Hospital of Sun Yat-sen University, Guangzhou, China

**Keywords:** decision aid, breast reconstruction, patient satisfaction, clinical outcomes, regret

## Abstract

**Objective:** To design, develop, and evaluate via focus group a preconsultation decision aid to improve patient satisfaction for breast reconstruction. **Methods:** The design of the decision aid was based on perceived patient needs, literature, existing decision aids, and current standard of breast cancer reconstruction treatment and consultation at Stanford. Our decision aid was designed to (1) reducing fear of the unknown in patients via providing a knowledge base that they can rely on, (2) helping patients identify their key breast reconstruction concerns, (3) addressing common patient concerns, (4) providing a framework to help patients identify the treatment option that may be right for them, and (5) promoting shared decision making. Physicians were consulted on the decision aid, following which a focus group was conducted for patient feedback. **Results:** Interviewed patients (*n* = 12) were supportive of the decision aid initiative. Participants were especially pleased with the side-by-side comparison of surgical options and the parsimonious way information was represented. All patients before undergoing reconstruction (*n* = 3) requested the decision guide to reference at home. All interviewed patients believed information level was “about right.” **Conclusions:** Decision aid was well received by patients in the focus group. As the initiative is for quality improvement, we saw no need to further delay the distribution of the decision aid. A pilot study will be conducted to evaluate whether our decision aid has an effect on patients’ decision regret, stress, and anxiety.

Breast cancer affects 1 in 8 women in the United States during their lifetime. The cancer is unique in that breasts are an important part of female gender identity and self-image. As such, treatment decisions for breast cancer and reconstruction can be complicated by psychological and practical—lifesaving—considerations. Patients are typically asked to concurrently make 2 different decisions. The first decision relates to the treatment and/or removal of cancer tissue. The second decision addresses whether they want reconstruction, and, if so, what type of reconstruction and when to have it.

Our candidates undergoing reconstruction will have had their breast partially/completely removed. Studies show that mastectomy and lumpectomy alone can result in increased levels of depression,^1^ distortion of body image,^2^^-^5 lower self-perceived sexual attractiveness,^6^^,^^7^ lower sexual satisfaction,^6^^,^^7^ and a negative impact on sexual life.^6^ Because of these negative effects, reconstructive options are presented to the patient following their oncological consultations. Indeed, reconstruction can help negate some of these issues; studies show that breast reconstruction can improve physical function, health perception, vitality, social function, emotion, mental health, and self-esteem in patients after mastectomy/lumpectomy.^8^ Women who opted for a delayed reconstruction reported that they did so to feel more feminine, look natural, wear different clothes, and get their lives back.^9^

While it may seem that the pairing of removal and reconstruction of the breast should negate the sentiment involved in the first decision, there remain cases of patients refusing mastectomy and lumpectomy as part of their cancer treatment due to image concerns. In addition, many patients report feeling overwhelmed by the decision making required throughout the process whereas others seem bogged down on one aspect of either decision (removal or reconstruction) and do not seem to fully think through the other. There is likely a heavy emotional and cognitive burden on patients with breast cancer as they face vulnerability not only in their mortality but also in their sexuality and gender-defining tissue(s). Understanding that the 2-step decision is difficult for many of our patients, we wanted to provide them with a preconsultation decision aid.

Decision aids have been effectively used in conjunction with or following consultations. Studies show that they can help facilitate patients’ cognitive strategies and change emotional processes,^10^ reduce decision regret,^7^^,^^11^^-^14 and improve health literacy^12^^-^14 in patients with breast cancer. However, upon review of existing decision aids, we felt that they primarily attempted to supply patients with a medical-grade education. These did not suite our purposes, as decision aids with too much information may increase confusion and lead to distress.^15^ In addition, we did not think that existing decision aids adequately addressed patients’ state of stress, concerns, fears, and vulnerability postdiagnoses and preconsultation. We wanted our decision aid to provide patents with a preliminary understanding of breast reconstruction, forming a base that we could build upon during the initial consultation.

While existing decision aids focused on providing patients with medical-grade education, our decision aid was focused on helping patients make their decision. To facilitate this, we had the following goals in mind: (1) reducing fear of the unknown in patients by providing a knowledge base that they can rely on, (2) helping patients identify their key breast reconstruction concerns, (3) addressing common patient concerns, (4) providing a framework to help patients identify the treatment option that may be right for them, and (5) to promote shared decision making. We hypothesize that our patients would find such a “decision guide” helpful in their decision making.

The Stanford institutional review board exempted this study, which is a quality improvement initiative, from standard review. Per Stanford institutional review board policy for exempted studies, we are still able to share findings.

## METHODS

A decision aid created by Lam et al at Hong Kong University was shown to be effective in reducing decision regret in patients with breast cancer in a Chinese population.^7^^,^^16^ Its format, which was based on a minimalistic design, resembled what we wanted for our decision aid. The key design considerations for our decision aid were based on analysis of the structure of the guide and the functions of its components. Our key considerations were succinct information, emotional support, and decisional support.

### Succinct information

**Figure 1:**
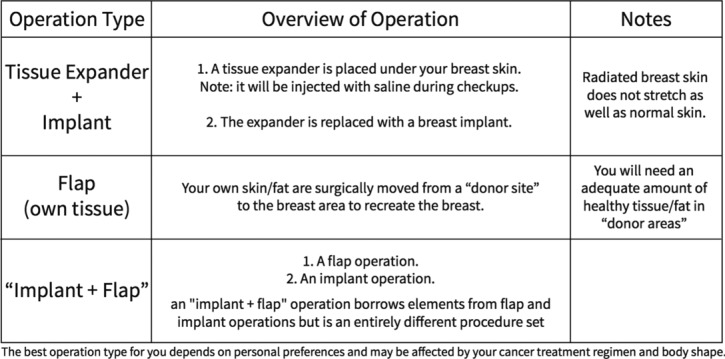
Example of text.

**Figure 2:**
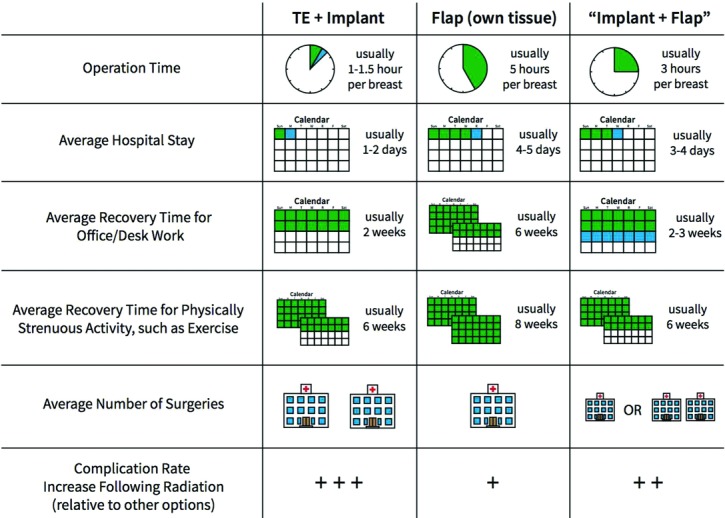
Comparison table.

**Figure 3:**
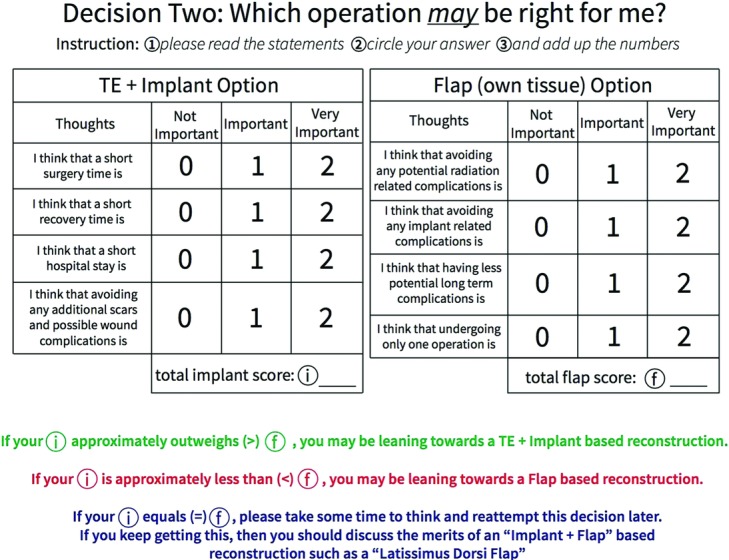
Decisional component.

We summarized all the information presented by our physicians during their initial patient consults. The patient could rely on this information. Content was curated to provide patients enough understanding so that they could ask informed questions during consultation and that they would present in consultation with a reasonable basis of information on hand. Lay terms and easy-to-understand words/phrases were used whenever possible (Fig 1). Tables, images, and icons to visually represent differences were used for comparisons (Fig 2). Important considerations were paraphrased into simple questions and built into our decisional component (Fig 3).

### Emotional support

Common concerns of our patients were combined with those reported in a study.^17^ These aggregate concerns were addressed via clear, one-sentence, large-font, and prominent statements such as “No matter which breast reconstruction option you choose, your chance of cancer resurgence is not changed.” Such statements serve 3 primary purposes. The first is to allay some of the stress and anxiety patients may feel. The second is to help take certain concerns out of their active considerations (eg, concerns about different cancer prognosis are negated by the statement), thereby reducing cognitive load. Third, to help better vocalize patient concerns so that they can identify those important to them and discuss them with their physician.

### Decisional support

**Figure 4:**
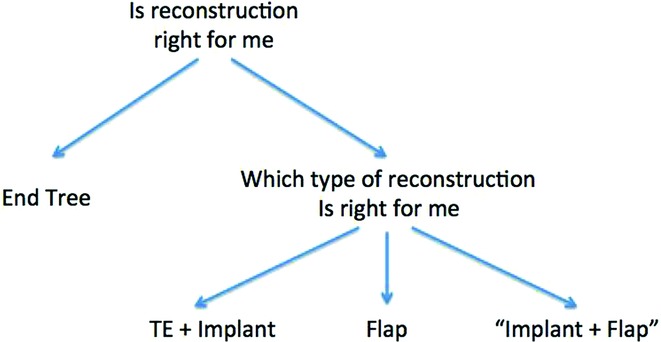
Decision tree logic outline. TE indicates tissue expander.

Questions our physicians ask patients to help them make a decision on treatment were paraphrased into simple questions and displayed in our decisional component (Fig 3). The decisional component is set up to help patients form a preliminary decision to be discussed with their physician during their appointment. The decisional supports were also set up so that decisions branch as indicated in Figure 4.

Our design and content iterations were presented to departmental physicians for review and updated accordingly. This iteration was then analyzed in a focus group with current patients—both prereconstruction and postreconstruction. Patients were asked a series of predetermined open-ended questions (Table 1). The interview questions were split into 2 sections: those to be answered before seeing the decision aid, and those to be answered after seeing the decision aid. Patients had the option of not providing answers but were encouraged to answer these questions. An open-ended tally system was used so that patients could provide more than 1 comment per question. The final decision aid was updated to address patient responses. Participation was not mandatory. Interviews were kept within 15 minutes.

## RESULTS

### Postoperative patients

Answers to questions provided before seeing our decision aid were as follows. Postoperative patients indicated that the following were most important to them when they were considering their reconstructive options: results/prognosis (4 of 9 postoperative patients [4/9]), complications (2/9), patient stories/experience (2/9), understanding choices (2/9), no choice due to body phenotype (2/9), and beating cancer (1/9). When asked what they wish they knew before deciding on reconstruction, only recovery pain (1/9) and “what questions should I ask my physician” (1/9) were indicated.

Answers to questions provided after seeing our decision aid were as follows. They indicated that the component of the decision guide they most liked were the comparison section (8/9), specific information (2/9), and decisional help (1/9). When asked what they like least, they indicated a component of the guide needed clarification (5/9), section(s) were irrelevant to specific patient (2/9), and too much information (1/9). When asked what we were missing that may help other patients, they responded with past patient experience (2/9), appearance or results (1/9), and specific questions to ask physician (1/9).

### Preoperative patients

Answers to questions provided after seeing our decision aid were as follows. Preoperative patients most valued our facilitation of patient choice (3 of 3 preoperative patients [3/3]) and that the decision aid was helpful to thinking through choices (1/3). They least liked components of the guide that they thought needed clarification (3/3). Preoperative patients were not asked questions from the other section, as they were not applicable.

### General

When asked to circle whether the information provided was “too much,” “about right,” “too little,” all patients (12/12) circled “about right.” No patient declined to participate in the focus group.

## DISCUSSION

Our decision aid was created with the following major sections: breast reconstruction options, overview and details of differences using a simple comparison structure, comparison of complications, components to help patients make a decision, and validated online material for additional information. Of the elements mentioned by postoperative patients to have been important during their consideration of reconstruction options, only patient experiences were not touched upon by our decision aid. This was maintained into the final draft, as we felt patient experiences can vary greatly and providing vignettes may seed inaccurate expectations.

In our study, 8 of 9 postoperative patients deemed the comparison section as the one they most liked. This may be due to having the essence of the differences between the operative types clearly delineated. Many postoperative patients commented that they wish they had access to our decision aid when making their reconstructive decision. This is consistent with research showing that comparisons structures help improve patient decision making.^18^^-^19 Five of 9 postoperative patients and all of the preoperative patients indicated that portions of the guide needed clarification. This referred predominately to items of the comparison section. There were some images and textual representations that were not perfectly clear and were subsequently updated. All of the preoperative patients indicated that our guide would help them think through their decision making. Interestingly, all preoperative patients asked for a copy of the decision aid to bring home and 1 patient followed up with us when she could not find the digital copy we sent.

All patients informed us that the information provided was “about right.” Interestingly, the 1 patient who indicated that the guide may have had “too much information” when asked for complaints answered “about right” when asked about information load in the guide. We believe that these responses were due to the design of the guide to be as succinct and simplistic as possible. Notably, the guide is adjusted to be understandable at the lower end of the educational/English proficiency spectrum via heavy use of graphics and simple-to-understand verbiage. This is because under duress, people's comprehension is usually compromised. We attribute the decision aid's warm reception due to the heavy use of graphics, simplistic verbiage, and decisional elements.

We used images whenever possible, as past studies show that images and diagrams were easier for patients to comprehend than the text.^20^^-^21 They may also increase comprehension since people try to interpret images when they see them, therefore internalizing the presented information more than they would otherwise. We used graphics instead of numbers whenever possible as well. For example, instead of saying X weeks in the hospital, we filled a calendar with the expected hospital stay and recovery time so that patients could fully appreciate the length. Simple text and prolific use of graphical representations make the decision aid easier to understand and will likely allow us to educate our patients more so than decision aids full of medical terms and superfluous details.

The verbiage was simplified as best we could without compromising meaning. With the exception of complications, we avoided medical terminology where possible. This allowed the decision aid to be more easily understood by lay individuals. We also provided reputable online resources for patients who wanted to generate a higher level of understanding of their reconstructive options. Only 1 patient (1/12) indicated that the links would be useful to her; everyone else seemed sufficiently happy with the information in the guide.

Decisional elements were built into the guide. Apart from our decision tree structure, the questions within the decisional elements (Fig 3) were personalized to the patient. For example, “I think that not having to wear a prosthesis is…” was used instead of “How important is it for you to not wear a prosthesis” because the “I” in the former question better poses the question to the individual whereas the use of “you” tends to externalize it. We wanted the patients to visualize themselves in the situation and to think of the answers from a personal viewpoint, elicited by “I” instead of a general viewpoint. The questions in the decisional component can be difficult, so we limited patient cognitive load to answering 1 question at a time through our score tally system.

One limitation of utilizing focus group testing is the relatively low number of patients; with a higher number of patients, we might have been able to get a better understanding of patient needs. Also, our interview system is not as rigorous as a controlled study. Patients may feel an innate need to be supportive of their providers; therefore, to counteract this, interview questions were written in a manner so as to elicit a negative response. However, the findings from our pilot study showed that we had a very strong positive response from patients in support of the decision aid and that it might be very welcome by patients. Ultimately, the decision aid may help patients and surgeons determine together the best choice of breast reconstruction for the patient.

## CONCLUSION

Our goal was to create a decision aid to help guide patients through their choices before their consultation with a physician—allowing patients to ask pertinent questions during consultation and for our physicians to more easily build upon patients’ knowledge of reconstruction. This was done via focusing on (1) reducing fear of the unknown in patients via providing a knowledge base that they can rely on, (2) helping patients identify their key breast reconstruction concerns, (3) addressing common patient concerns, (4) providing a framework to help patients identify the treatment option that may be right for them, and (5) promoting shared decision making.

Our decision guide was well received by both our preoperative and postoperative reconstruction patients. On the basis of the interviews, we conclude that our patients would find such a decision guide helpful in their decision making. As the initiative is for quality improvement, we saw no need to further delay the guide's distribution to patients and we are launching a pilot program aimed at evaluating the guide's effect on decision regret, stress, and anxiety.

## Figures and Tables

**Table 1:** Focus interview questions

Before looking over decision guide
1. When you were making your decision about breast reconstruction, what were the most important considerations?
2. Is there any information you wish you knew before deciding on reconstruction?
After looking over decision guide
1. What component(s) of the decision guide did you like the most?
2. What component(s) of the decision guide did you like the least?
3. What do you think we are missing that would be helpful to patients?
4. Did you find anything confusing or unclear?
5. Do you think the information provided is too much, about right, too little?
6. Any other comments or suggestions?
